# The Role of Fetal Renal Artery Doppler in Predicting Intrauterine Growth Restriction: A Case-Control Study

**DOI:** 10.7759/cureus.106775

**Published:** 2026-04-10

**Authors:** Sneha D N, Anil K Sakalecha, Guru Yogendra Muthyal, Krishna G K, Rakesh Bhixavatimath, Vimarshitha P

**Affiliations:** 1 Radiodiagnosis, Sri Devaraj Urs Medical College, Kolar, IND; 2 Radiology, Sri Devaraj Urs Medical College, Kolar, IND; 3 Obstetrics and Gynecology, Sri Devaraj Urs Medical College, Kolar, IND

**Keywords:** fetal growth restriction, fetal renal artery doppler, intrauterine growth restriction, prenatal surveillance, pulsatility index, resistive index, systolic/diastolic ratio

## Abstract

Background: Fetal growth restriction (FGR) is a clinically significant condition linked to increased perinatal morbidity, stillbirth, and adverse long-term neurodevelopmental outcomes. Conventional Doppler assessment using the umbilical artery (UA) and middle cerebral artery (MCA) provides well-established markers of placental insufficiency and fetal cerebral adaptation. However, these parameters often identify fetal compromise only in relatively advanced stages of disease. Consequently, a significant number of high-risk pregnancies complicated by FGR may not be detected early when relying solely on UA/MCA Doppler assessments. Evaluation of the fetal renal artery using Doppler ultrasonography has been proposed as a potential early indicator of systemic hemodynamic adaptation to chronic fetal hypoxia. This study aimed to evaluate fetal renal artery Doppler indices in FGR and assess their utility as early markers of fetal compromise and risk stratification.

Methods: A prospective observational study was conducted at RL Jalappa Hospital, Tamaka, Kolar, on 60 singleton pregnancies diagnosed with FGR, compared with gestational age-matched appropriate-for-gestational-age (AGA) controls from September 2025 to December 2025. Doppler velocimetry of the fetal renal artery was performed to measure pulsatility index (PI), resistive index (RI), and systolic/diastolic (S/D) ratio. Simultaneous assessment of UA and MCA Doppler was performed. Statistical comparisons were made using Student’s t-test or Mann-Whitney U test.

Results: Renal artery PI, RI, and S/D ratios were significantly elevated in FGR fetuses compared with AGA controls (p < 0.001), indicating increased downstream resistance and reduced diastolic flow. Renal artery PI demonstrated good diagnostic performance in detecting FGR, with a sensitivity of 86.7%, a specificity of 83.3%, and an overall accuracy of 85% at the optimal cutoff value. These findings indicate that renal artery PI is a reliable parameter for identifying fetuses at risk of growth restriction. PI may substantially influence clinical decisions, such as increasing monitoring frequency or advancing delivery timing when thresholds for potential compromise are approached. Renal Doppler indices correlated with the severity of growth restriction and the presence of oligohydramnios, suggesting early systemic adaptation prior to overt UA or MCA abnormalities.

Conclusions: Fetal renal artery Doppler indices are sensitive markers of early hemodynamic compromise in FGR. Incorporating renal Doppler into standard surveillance protocols may serve as a complementary parameter for evaluating FGR; however, its role in early detection and clinical decision-making requires further validation. Renal Doppler assessment should be considered when UA Doppler results are normal, but FGR is still suspected.

## Introduction

Fetal growth restriction (FGR) is a pathological condition in which a fetus fails to achieve its genetically determined growth potential, most often due to placental insufficiency and compromised uteroplacental blood flow [1,2]. FGR remains a major global health problem, contributing to perinatal morbidity, stillbirth, and long-term neurodevelopmental impairment [1,3]. Globally, an estimated 23-32 million infants are born small-for-gestational-age (SGA) each year, many with underlying growth restriction, and these infants account for a significant proportion of neonatal deaths [3,4]. In 2020, over 23 million live births were SGA, and FGR contributed to nearly two million stillbirths worldwide, highlighting its burden, especially in low and middle-income countries where limited prenatal care and maternal malnutrition are common. In contrast, the prevalence of FGR in high-income countries is lower, about 3%-9%, reflecting differences in maternal health, nutrition, and antenatal care infrastructure [5,6].

Early detection of fetal compromise is essential to improve perinatal outcomes [2,7]. Doppler velocimetry of the umbilical artery (UA) and middle cerebral artery (MCA) is routinely used to monitor placental resistance and cerebral redistribution, guiding surveillance and timing of delivery. However, these conventional parameters often reflect hemodynamic compromise at advanced stages [7,8]. Emerging evidence suggests that additional vascular beds, such as the fetal renal artery, may provide earlier and complementary information about systemic fetal adaptation [9,10]. The fetal renal artery, normally a low-resistance vascular bed, undergoes vasoconstriction in response to chronic hypoxia and placental insufficiency. This vasoconstriction reduces urine output, leading to measurable increases in the pulsatility index (PI), resistive index (RI), and systolic/diastolic (S/D) ratio, making it a potential early marker of compromise [10]. Given its noninvasive, reproducible nature, renal artery Doppler may serve as a practical adjunct to conventional UA and MCA assessment, offering potential for early risk stratification and improved management of growth-restricted pregnancies [9,10].

The present study aims to evaluate fetal renal artery Doppler indices in fetuses with FGR compared with appropriate-for-gestational-age (AGA) controls and to assess their utility as early markers of fetal hemodynamic compromise.

## Materials and methods

Study design and setting

This hospital-based prospective case-control study was conducted in the Department of Radiology at a tertiary care teaching hospital over a three-month period. The study aimed to evaluate the role of fetal renal artery Doppler indices in predicting intrauterine growth restriction (IUGR). A total of 60 participants were consecutively recruited during the study period from December 2025 to February 2026. This study was approved by the Institutional Ethics Committee of Sri Devaraj Urs Medical College, Kolar, India (approval no: SDUAHER/R&D/CEC/SDUMC-PG/287/NF/2025-26). The study adhered to the principles of the Declaration of Helsinki, and written informed consent was obtained from all participants.

Study population and eligibility criteria

Inclusion Criteria

Pregnant women with viable singleton gestations referred for fetal growth assessment and Doppler evaluation between 28 and 36 weeks of gestation were enrolled consecutively. Fetuses with structurally normal anatomy on ultrasonography and mothers providing informed consent were included.

Based on estimated fetal weight (EFW), calculated according to Hadlock’s biometric formula, fetuses were categorized into two groups: IUGR (EFW <10th percentile for gestational age) and AGA (EFW between the 10th and 90th percentiles), constituting the case and control groups, respectively.

Exclusion Criteria

Pregnancies with multiple gestations, major congenital or chromosomal anomalies, maternal chronic renal or systemic disorders affecting uteroplacental or fetal hemodynamics, severe oligohydramnios unrelated to placental insufficiency, pregnant women treated with aspirin, or inadequate acoustic windows preventing reliable Doppler acquisition were excluded to minimize confounding and measurement bias.

Imaging procedure and Doppler assessment

All examinations were performed using standardized obstetric ultrasonography using the GE-VOLUSON E6 RADIANCE BT 19 ultrasound system (GE HealthCare, Zipf, Austria). Fetal biometry included biparietal diameter, head circumference, abdominal circumference, and femur length for estimation of fetal weight. Color Doppler was used to identify the fetal right renal artery at its origin from the abdominal aorta, followed by pulsed-wave Doppler spectral analysis.

Waveforms were recorded during fetal quiescence to minimize motion artifacts, and angle correction was maintained below 30°. Doppler indices, including PI, RI, and S/D ratio, were automatically calculated by the machine. The mean of three consecutive uniform waveforms was used for analysis. Renal artery Doppler parameters were compared between the IUGR and AGA groups. Blinding of sonographers to group allocation was not feasible, which may introduce observer bias and could affect measurement reproducibility.

Doppler assessment is inherently operator-dependent; however, all examinations were performed using a standardized protocol by experienced radiologists to minimize interobserver variability. Interobserver variability was not formally assessed in this study, which may limit the evaluation of measurement reproducibility.

Study Endpoints

The primary endpoint was the difference in fetal renal artery Doppler indices between IUGR and AGA fetuses. Secondary endpoints included assessment of the diagnostic performance of these indices for identifying IUGR using receiver operating characteristic (ROC) curve analysis.

Statistical analysis

Data were entered into Microsoft Excel (Microsoft Corporation, Redmond, WA) and analyzed using IBM Statistical Package for the Social Sciences Statistics version 22 (IBM Corp., Armonk, NY). Continuous variables were expressed as mean ± standard deviation or median with interquartile range, depending on distribution, while categorical variables were presented as frequencies and percentages. Intergroup comparisons used the independent samples t-test or Mann-Whitney U test for continuous variables and the chi-square or Fisher’s exact test for categorical variables. The sample size of 60 participants (30 per group) was calculated based on an expected moderate effect size in renal artery Doppler indices between the groups, with 80% power and a 5% level of significance. ROC curve analysis determined diagnostic accuracy and optimal cutoff values. Sensitivity, specificity, positive predictive value, and negative predictive value were calculated. Multivariate logistic regression identified independent predictors of IUGR. A p value of <0.05 was considered statistically significant.

## Results

Study population

A total of 60 pregnant women with singleton gestations were included in the analysis. Based on EFW percentiles, 30 fetuses were classified as the IUGR group and 30 as AGA controls. All participants completed ultrasound and Doppler assessment and were included in the final statistical analysis.

Maternal age and gestational age at examination were comparable between the groups (p > 0.05). Fetal abdominal circumference, EFW, and amniotic fluid index were significantly lower in the IUGR group compared with controls (p < 0.05). Table 1 summarizes the baseline characteristics of maternal age, gestational age at scan, fetal abdominal circumference, EFW, and amniotic fluid index in the IUGR group and AGA the control group.

**Table 1:** Baseline demographic and obstetric characteristics of the study population Values are expressed as mean ± standard deviation or frequency (%). Continuous variables were compared using the independent-samples t-test ^*^p < 0.05 indicates statistical significance IUGR: intrauterine growth restriction

Variable	IUGR (n = 30)	Control (n = 30)	t value	p value
Maternal age (years)	27.1 ± 4.5	26.4 ± 4.1	0.63	0.52
Gestational age at scan (weeks)	32.8 ± 2.4	33.1 ± 2.2	0.5	0.61
Abdominal circumference (mm)	258 ± 18	295 ± 20	7.54	<0.001^*^
Estimated fetal weight (g)	1,580 ± 210	2,210 ± 260	10.28	<0.001^*^
Amniotic fluid index (cm)	8.1 ± 2.0	10.3 ± 2.4	3.85	0.01^*^

Fetal renal artery Doppler indices (PI, R1, and S/D ratio) revealed significantly increased vascular resistance and reduced diastolic flow in IUGR fetuses compared with controls, indicating increased renal vascular resistance in growth-restricted fetuses. Table 2 presents the comparison of fetal renal artery Doppler parameters between the IUGR and control groups, demonstrating higher PI, RI, and S/D ratios in the IUGR group with statistically significant differences.

**Table 2:** Comparison of fetal renal artery Doppler parameters between IUGR and control groups Values are expressed as mean ± SD. Comparisons were performed using an independent-samples t-test and ANOVA ^*^p < 0.05 indicates statistical significance PI: pulsatility index; RI: resistive index; S/D: systolic/diastolic; SD: standard deviation; IUGR: intrauterine growth restriction; ANOVA: analysis of variance

Parameter	IUGR (mean ± SD)	Control (mean ± SD)	Mean difference	t value	F value	p value
PI	2.18 ± 0.34	1.61 ± 0.29	0.57	6.99	48.86	<0.001^*^
RI	0.86 ± 0.05	0.73 ± 0.06	0.13	9.12	83.17	<0.001^*^
S/D ratio	5.21 ± 0.92	3.52 ± 0.74	1.69	7.8	61.47	<0.001^*^

**Figure 1:**
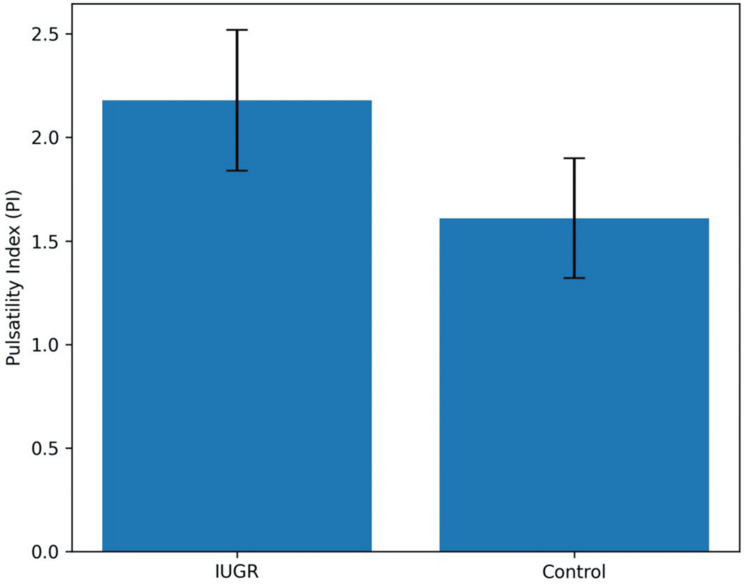
Comparison of fetal renal artery PI between IUGR and control groups Bar graph demonstrating mean fetal renal artery PI in IUGR fetuses and appropriate for age (control) fetuses. Bars represent mean values, and black error bars indicate ±SD. The IUGR group shows significantly higher PI, reflecting increased renal vascular resistance and reduced diastolic perfusion (p < 0.001) PI: pulsatility index; IUGR: intrauterine growth restriction; SD: standard deviation

Figure 1 illustrates the comparison of mean fetal renal artery PI between IUGR and control fetuses. The mean fetal renal artery PI in the IUGR group was significantly higher than in controls (2.18 ± 0.34 vs. 1.61 ± 0.29; p < 0.001), indicating increased renal vascular resistance; error bars in Figure 1 represent ±standard deviation.

Distribution of abnormal renal artery Doppler findings

Table 3 summarizes the frequency of abnormal fetal renal artery Doppler indices among the study groups, demonstrating a higher proportion of elevated PI, RI, and S/D ratio in the IUGR group than in controls.

**Table 3:** Frequency of abnormal renal artery Doppler indices in the study groups Values presented as a number (%). Comparisons were performed using the chi-square test. PI >95th percentile and RI >0.80 were considered abnormal. p < 0.05 indicates statistical significance IUGR: intrauterine growth restriction; PI: pulsatility index; RI: resistive index; S/D: systolic/diastolic

Parameter abnormality	IUGR (n = 30)	Control (n = 30)	χ² value	p value
Elevated PI	24 (80%)	5 (16.7%)	24.08	<0.001
Elevated RI	22 (73.3%)	6 (20%)	17.14	<0.001
Increased S/D ratio	23 (76.7%)	7 (23.3%)	17.07	<0.001

Diagnostic accuracy of renal artery Doppler indices

Diagnostic performance analysis demonstrated good discriminatory ability of renal artery Doppler indices for identifying IUGR. PI showed the highest overall diagnostic accuracy, followed by RI and the S/D ratio. Table 4 summarizes the diagnostic performance of fetal renal artery Doppler indices for predicting IUGR, including sensitivity, specificity, positive predictive value, negative predictive value, and overall accuracy.

**Table 4:** Diagnostic performance and accuracy of renal artery Doppler indices for prediction of IUGR Cutoff values were determined using Youden’s index PPV: positive predictive value; NPV: negative predictive value; PI: pulsatility index; RI: resistive index; S/D: systolic/diastolic; IUGR: intrauterine growth restriction

Parameter	Cutoff	Sensitivity (%)	Specificity (%)	PPV (%)	NPV (%)	Accuracy (%)
PI	>1.90	86.7	83.3	84.4	85.7	85
RI	>0.80	83.3	80	81	82.7	81.7
S/D ratio	>4.5	80	76.7	78.4	78.3	78.4

**Figure 2:**
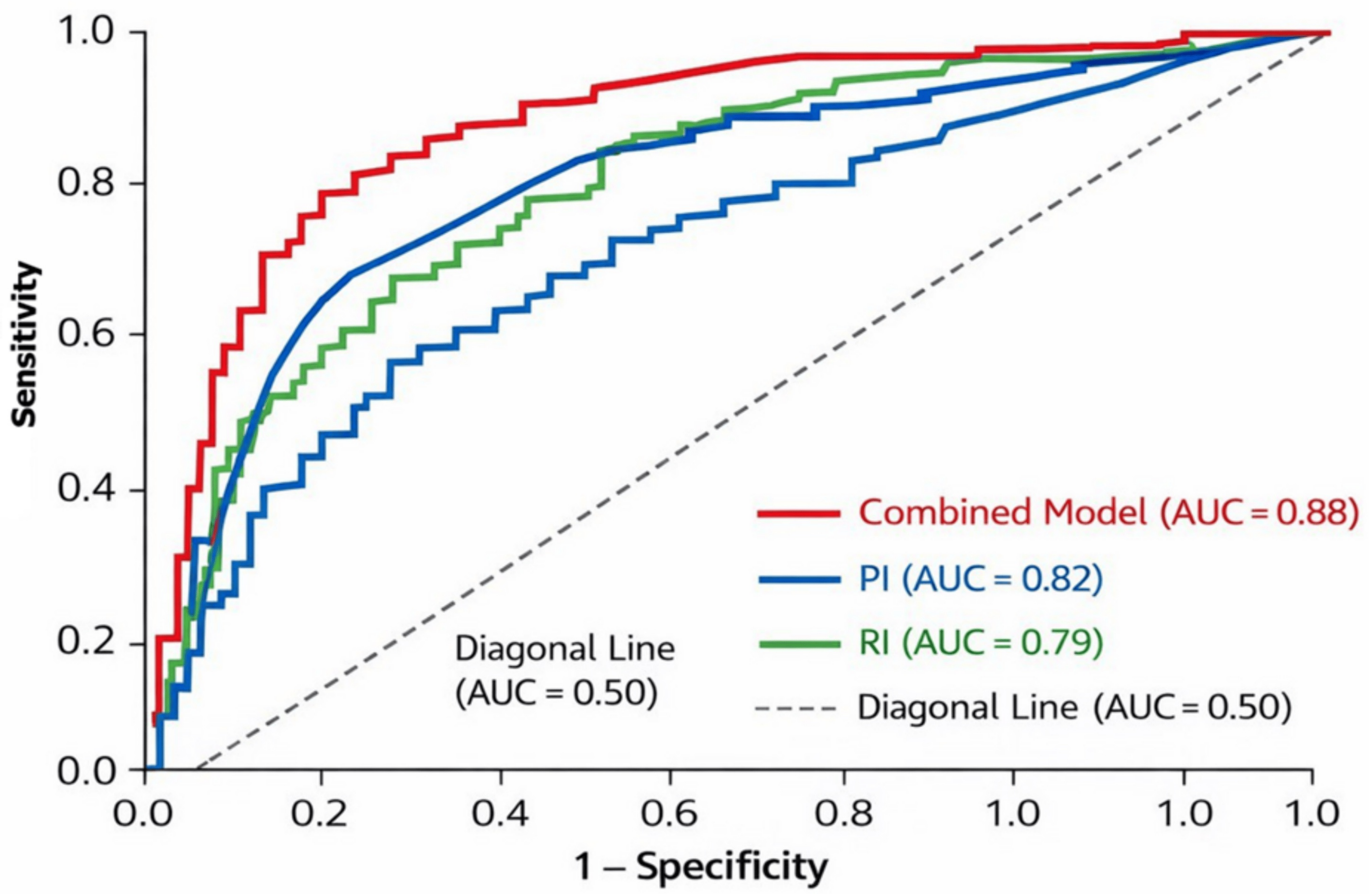
ROC curves of fetal renal artery Doppler indices for prediction of intrauterine growth restriction ROC curves showing the diagnostic performance of fetal renal artery Doppler indices for predicting intrauterine growth restriction. PI demonstrated the highest accuracy, followed by the RI and the systolic/diastolic ratio. The diagonal line indicates no discrimination (AUC = 0.50) ROC: receiver operating characteristic; AUC: area under the curve; PI: pulsatility index; RI: resistive index

Figure 2 shows the ROC curve analysis of fetal renal artery Doppler indices for predicting IUGR, indicating good discriminative ability of these parameters.

Predictors of IUGR

Table 5 shows the multivariate logistic regression analysis identifying independent predictors of IUGR, including elevated renal artery PI, RI, and low AFI. In multivariate logistic regression, elevated renal artery PI and RI remained independently associated with IUGR after adjustment for gestational age and amniotic fluid index. Elevated PI showed the strongest association with IUGR (OR = 4.2; 95% CI: 1.8-9.7).

**Table 5:** Multivariate logistic regression analysis for predictors of IUGR Logistic regression adjusted for gestational age and amniotic fluid index p < 0.05 indicates statistical significance CI: confidence interval; AFI: amniotic fluid index; PI: pulsatility index; RI: resistive index; IUGR: intrauterine growth restriction

Variable	Odds ratio	95% CI
Elevated PI	4.2	1.8-9.7
Elevated RI	3.6	1.5-8.4
Low AFI	2.1	0.9-4.8

**Figure 3:**
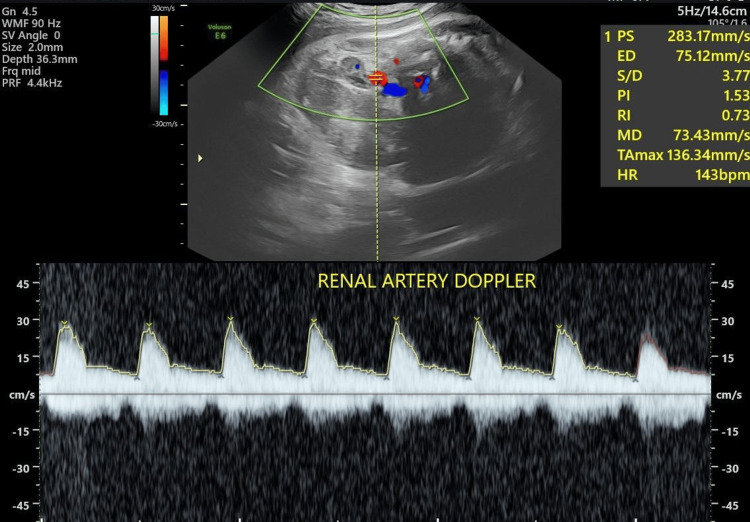
Color Doppler spectral waveform of the fetal renal artery in an AGA control fetus Color Doppler-guided spectral Doppler evaluation of the fetal renal artery demonstrating a normal arterial waveform with preserved diastolic flow. The measured Doppler indices include a PI of 1.5, a RI of 0.7, and an S/D ratio of 3.7, consistent with normal renal vascular resistance in an appropriately grown fetus PI: pulsatility index; RI: resistive index; S/D: systolic/diastolic; AGA: appropriate-for-gestational-age; PS: peak systolic velocity; ED: end-diastolic velocity; MD: minimum diastolic velocity; HR: heart rate; TAmax: time-averaged maximum velocity; WMF: wall motion filter; SV: sample volume; PRF: pulse repetition frequency

**Figure 4:**
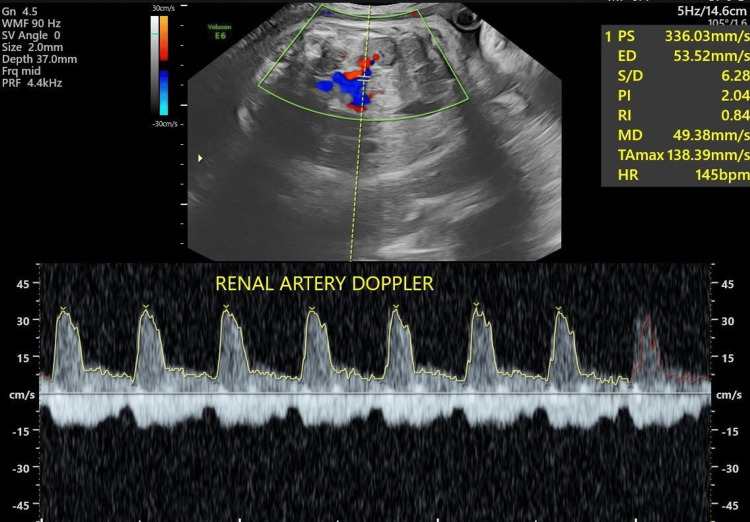
Color Doppler spectral waveform of the fetal renal artery in an IUGR fetus Color Doppler-guided spectral Doppler evaluation of the fetal renal artery demonstrating increased vascular resistance with reduced diastolic flow. The measured Doppler indices include a PI of 2.0, an RI of 0.8, and an S/D ratio of 6.2, consistent with increased renal arterial resistance in intrauterine growth restriction PI: pulsatility index; RI: resistive index; S/D: systolic/diastolic; AGA: appropriate-for-gestational-age; PS: peak systolic velocity; ED: end-diastolic velocity; MD: minimum diastolic velocity; HR: heart rate; TAmax: time-averaged maximum velocity; WMF: wall motion filter; SV: sample volume; PRF: pulse repetition frequency

Representative spectral Doppler waveforms of the fetal renal artery are shown in Figures 3, 4, demonstrating normal Doppler indices in an AGA fetus and increased vascular resistance in an IUGR fetus.

## Discussion

FGR remains a major contributor to perinatal morbidity and mortality worldwide, affecting approximately 5%-10% of pregnancies and accounting for a disproportionate share of stillbirths, neonatal intensive care admissions, and long-term neurodevelopmental impairment [1,2]. Early identification of fetal hemodynamic compromise, therefore, becomes essential for obstetric surveillance and timing of delivery [3]. Placental insufficiency leads to progressive redistribution of fetal circulation, commonly described as the “brain-sparing effect,” characterized by preferential perfusion of vital organs at the expense of peripheral vascular beds [4-6]. Although umbilical and MCA Doppler indices are well-established markers of placental and cerebral adaptation, increasing evidence suggests that peripheral organs, particularly the kidneys, undergo vasoconstrictive changes in response to chronic hypoxia [7,8].

Physiologically, the fetal renal circulation represents a low-resistance vascular bed. In the setting of placental insufficiency, sympathetic activation and stimulation of the renin-angiotensin-aldosterone system increase renal vascular resistance, resulting in reduced diastolic flow and elevation of Doppler indices such as PI, RI, and S/D ratio [9-11]. In the present study, fetuses with FGR demonstrated significantly higher renal artery PI, RI, and S/D ratios compared with AGA controls. These findings are consistent with earlier reports showing increased renal vascular impedance in growth-restricted fetuses [12,13]. Subsequent Doppler-based studies confirmed that reduced renal blood flow can be measured noninvasively and correlates with disease severity [14,15]. The statistically significant elevation of renal artery PI observed in our cohort suggests heightened downstream resistance and reduced diastolic flow. Similar observations were reported by Sadat Jamal and Modarresi, who noted that renal PI had comparable predictive performance to UA Doppler for identifying compromised fetuses [16]. Likewise, Al-Ghazali et al. reported that renal vasoconstriction reflects systemic fetal adaptation and correlates with adverse perinatal outcomes [17].

Beyond its independent diagnostic utility, renal artery Doppler should be interpreted in conjunction with established fetoplacental and cerebral indices to better characterize the sequence of hemodynamic deterioration in FGR. UA abnormalities typically reflect increased placental resistance, whereas MCA changes indicate cerebral vasodilation and advanced redistribution. Renal vasoconstriction, however, represents peripheral circulatory compromise and may occur either concurrently with or slightly earlier than cerebral centralization, thereby providing incremental staging information. Studies evaluating multivessel Doppler models have demonstrated that combining renal, umbilical, and cerebral parameters improves sensitivity for detecting fetuses at risk of decompensation compared with single-vessel assessment alone. This integrative approach supports the concept that FGR is a systemic cardiovascular disorder rather than isolated placental pathology, and comprehensive vascular profiling may allow more accurate timing of delivery and individualized surveillance strategies [18,19].

An important consideration is the incremental value of renal artery Doppler over conventional parameters such as UA and MCA Doppler. While UA Doppler reflects placental resistance and MCA Doppler indicates cerebral redistribution, renal artery Doppler provides insights into peripheral vascular adaptation. The observed elevation in renal artery indices in our study suggests that renal vasoconstriction may occur early in the course of fetal compromise, potentially offering additional information when UA and MCA parameters are within normal limits. Thus, renal artery Doppler may serve as a complementary tool rather than a replacement, enhancing the overall sensitivity of Doppler surveillance in FGR.

It is important to distinguish FGR from SGA fetuses. While SGA refers to fetuses with an EFW below the 10th percentile, not all SGA fetuses are pathologically growth-restricted. In the present study, classification was based on EFW criteria, which may include constitutionally small but otherwise healthy fetuses. This distinction should be considered when interpreting the results, as Doppler abnormalities are more closely associated with true placental insufficiency and fetal compromise.

From a prognostic standpoint, reduced renal perfusion contributes to oligohydramnios, which is independently associated with intrapartum distress, operative delivery, and increased neonatal intensive care admissions. Emerging evidence also suggests that chronic fetal hypoperfusion may influence long-term renal and cardiovascular development, highlighting that renal Doppler abnormalities may reflect both acute compromise and broader developmental consequences [18-20]. In our cohort, abnormal renal Doppler findings were associated with higher rates of adverse perinatal outcomes, supporting the clinical relevance of incorporating renal artery assessment into routine surveillance, particularly in resource-limited settings where rapid and reproducible tools are essential.

The strengths of this study include its prospective design, standardized Doppler acquisition protocol, and focused evaluation of fetal renal artery indices as early markers of systemic hemodynamic adaptation. This study has a few limitations that should be considered when interpreting the findings. The relatively small sample size and single-center design may limit the generalizability of the results. Additionally, Doppler assessment is inherently operator-dependent, and blinding of sonographers to group allocation was not feasible, which may introduce observer bias. Interobserver variability was not formally assessed, which may further affect measurement reproducibility. Furthermore, although renal artery Doppler indices were evaluated alongside routine parameters, a direct comparative analysis with established UA and MCA Doppler indices was not systematically performed, which limits assessment of their relative and incremental diagnostic value. These factors highlight the need for larger, multicenter studies with standardized protocols and comparative analyses to validate the findings. Overall, our findings support the concept that fetal renal artery Doppler indices reflect systemic hemodynamic compromise and serve as useful complementary markers in the evaluation of FGR; however, further longitudinal studies are required to establish their role in early identification and risk stratification.

## Conclusions

Fetuses with growth restriction demonstrated significantly elevated renal artery Doppler indices compared with controls (PI, RI, and S/D ratio; p < 0.01), indicating increased peripheral vascular resistance and altered circulatory dynamics. These findings suggest that renal artery assessment reflects systemic fetal adaptation to placental insufficiency and provides complementary information to conventional umbilical and MCA Doppler. Given its noninvasive, rapid, and reproducible nature, renal Doppler may serve as a practical adjunct in the evaluation and surveillance of pregnancies complicated by FGR. However, its role in early detection, risk stratification, prognostication, and clinical decision-making requires further validation in longitudinal studies.

## References

[1] Gordijn SJ, Beune IM, Thilaganathan B (2016). Consensus definition of fetal growth restriction: a Delphi procedure. Ultrasound Obstet Gynecol.

[2] Figueras F, Gratacós E (2014). Update on the diagnosis and classification of fetal growth restriction and proposal of a stage-based management protocol. Fetal Diagn Ther.

[3] Lawn JE, Ohuma EO, Bradley E (2023). Small babies, big risks: global estimates of prevalence and mortality for vulnerable newborns to accelerate change and improve counting. Lancet.

[4] Blencowe H, Krasevec J, de Onis M (2019). National, regional, and worldwide estimates of low birthweight in 2015, with trends from 2000: a systematic analysis. Lancet Glob Health.

[5] Baschat AA (2004). Doppler application in the delivery timing of the preterm growth-restricted fetus: another step in the right direction. Ultrasound Obstet Gynecol.

[6] Hecher K, Bilardo CM, Stigter RH (2001). Monitoring of fetuses with intrauterine growth restriction: a longitudinal study. Ultrasound Obstet Gynecol.

[7] Mari G, Deter RL (1992). Middle cerebral artery flow velocity waveforms in normal and small-for-gestational-age fetuses. Am J Obstet Gynecol.

[8] Gramellini D, Folli MC, Raboni S, Vadora E, Merialdi A (1992). Cerebral-umbilical Doppler ratio as a predictor of adverse perinatal outcome. Obstet Gynecol.

[9] Kingdom JC, Kaufmann P (1997). Oxygen and placental villous development: origins of fetal hypoxia. Placenta.

[10] Kiserud T (2005). Physiology of the fetal circulation. Semin Fetal Neonatal Med.

[11] Baschat AA, Gembruch U (2003). The cerebroplacental Doppler ratio revisited. Ultrasound Obstet Gynecol.

[12] Yoshimura S, Masuzaki H, Gotoh H, Ishimaru T (1997). Fetal redistribution of blood flow and amniotic fluid volume in growth-retarded fetuses. Early Hum Dev.

[13] Ebbing C, Rasmussen S, Godfrey KM, Hanson MA, Kiserud T (2009). Redistribution pattern of fetal liver circulation in intrauterine growth restriction. Acta Obstet Gynecol Scand.

[14] Fong K, Ryan ML, Cohen H, Amankwah K, Ohlsson A, Myhr T, Hannah M (1996). Doppler velocimetry of the fetal middle cerebral and renal arteries: interobserver reliability. J Ultrasound Med.

[15] Gembruch U (1996). Assessment of the fetal circulatory state in uteroplacental insufficiency by Doppler ultrasound: which vessels are the most practicable?. Ultrasound Obstet Gynecol.

[16] Sadat Jamal A, Modarresi M (2023). Renal artery Doppler in fetal sonography: a narrative review. Int J Reprod Biomed.

[17] Al-Ghazali W, Chita SK, Chapman MG, Allan LD (1989). Evidence of redistribution of cardiac output in asymmetrical growth retardation. Br J Obstet Gynaecol.

[18] Figueras F, Gratacos E (2014). Stage-based approach to the management of fetal growth restriction. Prenat Diagn.

[19] Baschat AA (2005). Arterial and venous Doppler in the diagnosis and management of early onset fetal growth restriction. Early Hum Dev.

[20] Magann EF, Chauhan SP, Doherty DA, Barrilleaux PS, Martin JN Jr, Morrison JC (2003). Predictability of intrapartum and neonatal outcomes with the amniotic fluid volume distribution: a reassessment using the amniotic fluid index, single deepest pocket, and a dye-determined amniotic fluid volume. Am J Obstet Gynecol.

